# Diffuse Large B Cell Lymphoma with Extensive Cutaneous Relapse

**DOI:** 10.1155/2015/137682

**Published:** 2015-09-20

**Authors:** Umit Yavuz Malkan, Gursel Gunes, Okan Yayar, Haluk Demiroglu

**Affiliations:** Department of Hematology, School of Medicine, Hacettepe University, 0100 Ankara, Turkey

## Abstract

Herein, we aimed to report a diffuse large B cell lymphoma (DLBCL) case that had extensive cutaneous relapse with no skin involvement previously. A 59-year-old man presented to hospital in April 2014 with fatigue, anorexia, fever, and anemia. Cervical lymph node biopsy revealed CD20+, BCL2+, MUM1+, BCL6+ high grade B lymphoproliferative neoplasm. After FISH investigation, he was diagnosed as DLBCL. He was given 7 cycles of R-CHOP and achieved remission. However, in November 2014, he had emerging skin lesions that cover nearly all of his body. A control PET-CT revealed diffuse cutaneous involvement. CD20+, BCL2+, MUM1+, BCL6+ high grade B cell lymphoma infiltration was detected with skin biopsy. He was diagnosed as relapse lymphoma, so 2 cycles of R-DHAP were given. There was no treatment response; therefore, R-ICE regimen was started. The patient had achieved second complete remission and his skin lesions were completely regressed. The involvement of skin with CD20+ cells after 7 cycles of rituximab therapy favors that there is a rituximab resistant disease which tends to involve the skin. To conclude, DLBCL may relapse extensively with cutaneous involvement and the best treatment option in these patients is salvage chemotherapy followed by autologous peripheral blood stem cell transplantation.

## 1. Introduction

Diffuse large B cell lymphoma (DLBCL) is the most common histologic subtype of non-Hodgkin lymphoma [1]. The majority of relapses occur during the first two years after completion of treatment [2, 3]. Relapses are usually symptomatic and rarely identified solely on the basis of routine imaging [4–6]. Extra nodal involvement of B cell lymphoma generally appears in gastrointestinal system followed by skin [7]. Skin involvement of B cell lymphomas could be either primary or secondary. Herein, we aimed to report a DLBCL case that had extensive cutaneous relapse with no skin involvement previously.

## 2. Case Report

**Figure 1:**
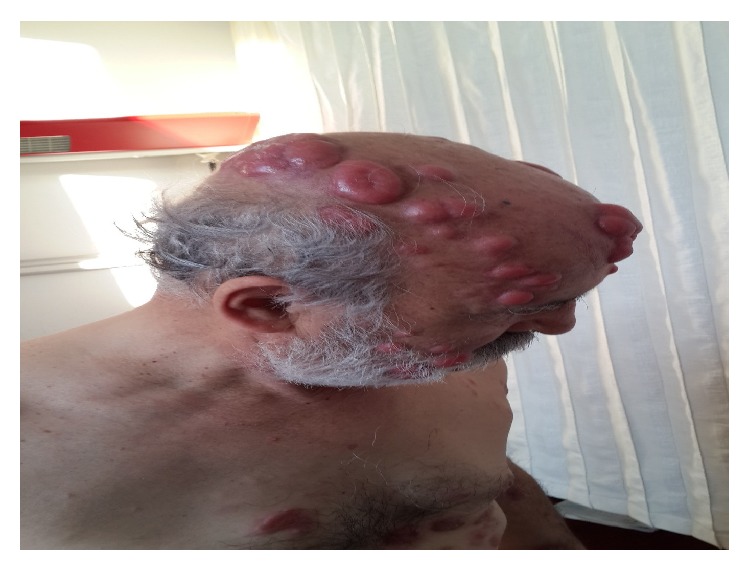
Prior to salvage treatment.

**Figure 2:**
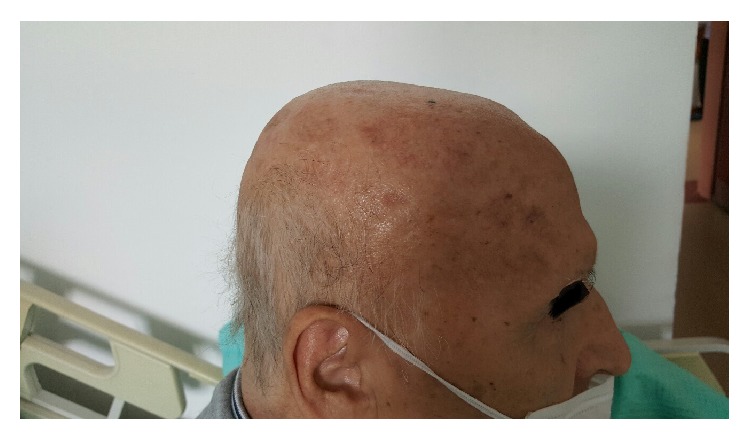
After salvage treatment.

A 59-year-old man presented to our clinic in April 2014 with fatigue, anorexia, fever, and anemia. Clinical examination revealed splenomegaly and cervical lymphadenopathies. Laboratory tests were reported as hemoglobin 9.1 gr/dL, white blood cell 6.1 × 10^3^/*μ*L, and platelets 430 × 10^3^/*μ*L with normal other biochemical values. HIV test was negative. Spleen size was found as 160 × 90 mm and lymphadenopathies around portal hilus were detected by abdominal USG. Cervical lymph node biopsy revealed high grade B lymphoproliferative neoplasm. Conventional cytogenetic analysis was performed; however it resulted in no metaphases. The FISH investigation has resulted in BCL2−, BCL6−, and MYC−. Immunohistochemical study has resulted in CD20+, BCL2+, MUM1+, BCL6+, and Tdt−. Ki-67 proliferation index of the patient was 90%. No disease involvement was detected in the bone marrow biopsy investigation. PET-CT was performed for disease staging. Involvement in spleen, bone marrow, cervical lymph nodes, bilateral axillary lymph nodes, thoracal lymph nodes, portal, para-aortic, paracaval, and bilateral inguinal lymph nodes, sternum, C2 vertebra, clavicula, acromion, and left humerus was detected, with SUV max value above 4. The patient was diagnosed as stage 4 diffuse large B cell lymphoma and 4 cycles of R-CHOP (rituximab, cyclophosphamide, adriamycin, vincristine, and prednisolone) regimen were given. In August 2014, cervical, thoracal, and abdominal CT revealed regression. R-CHOP regimen was given for 3 more cycles. The patient responded to the treatment well with complete resolution of all lymphadenopathies. However, 2 months after the completion of chemotherapy cycles in November 2014, the patient had emerging skin lesions that cover nearly all of his body. The lesions were painless and different in diameter with the biggest lesion reaching 5-6 cm (Figure 1). A control PET-CT revealed diffuse cutaneous involvement (SUV max 18.3) with axillary lymph node involvement (SUV max 14.8). Interestingly this time, spleen, inguinal nodes, and bone marrow involvement could not be detected. Biopsy was performed from skin lesion which was reported as CD20+, BCL2+, MUM1+, BCL6+ high grade B cell lymphoma infiltration. The patient also had foot drop which was considered to be secondary to vincristine. Electromyography revealed diffuse and severe axonal polyneuropathy and active myopathy in distal muscles. There was no involvement in cranial MR. He was diagnosed as relapsed lymphoma, so R-DHAP (rituximab, cisplatin, cytosine arabinoside, and dexamethasone) chemotherapy regimen was started. There was no treatment response after 2 cycles of R-DHAP; therefore, chemotherapy regimen has changed to R-ICE (rituximab, etoposide, carboplatin, and ifosfamide). With R-ICE treatment, the patient achieved second complete remission and his skin lesions were completely regressed (Figure 2). Autologous peripheral blood stem cell transplantation (PBSCT) was planned for our patient.

## 3. Discussion

Primary cutaneous lymphomas have better clinical course and prognosis [8, 9]. However, extensive secondary cutaneous involvement in systemic B cell lymphomas has been also reported in the literature [10, 11]. Very significant improvements were recorded in the management of DLBCL; however, it is still not usual to achieve cure with conventional treatment. The outcome of relapsed DLBCL largely depends on several factors such as fitness and chemosensitivity of the patient, eligibility for stem cell transplantation (SCT), IPI score at relapse, and time interval from previous chemotherapy. In the literature, it was stated that after the relapse of DLBCL the response rate following second line therapy (R-ICE or R-DHAP) was 63% [12]. Disease-free survival is very low in cases who reach second remission [13]. However, performing SCT in these patients would extend treatment response to two years [14]. Non-Hodgkin lymphomas generally relapse in the same involvement sites. In the literature, there are reports of cases who relapsed with CD20− skin involvement after rituximab therapy [15, 16]. Our case differs from these reports with CD20+ relapse after 7 cycles treatment with R-CHOP regimen. At the beginning of the treatment, our patient did not have skin involvement that excludes the diagnosis of primary cutaneous lymphoma. The involvement of skin with CD20+ after 7 cycles of rituximab therapy suggests that there is a resistant disease to rituximab which tends to involve the skin. Interestingly, disease relapse was not present in our patients' primary involvement sites, except axillary region. Moreover, disease relapse occurred in cutaneous region which did not have disease involvement primarily. To conclude, diffuse large B cell lymphomas may relapse extensively with cutaneous involvement and the best treatment option in these patients is salvage chemotherapy followed by autologous PBSCT.
